# Effects of Threshold Pressure Loading Exercises Applied to Inspiratory Muscles in Taekwondo Athletes on the Concentration and Utilization of Lactate

**DOI:** 10.5114/jhk/188542

**Published:** 2024-12-06

**Authors:** Murat Koç, Nazmi Sarıtaş, Betül Coşkun, Soner Akkurt

**Affiliations:** 1 Faculty of Sport Sciences, Erciyes University, Kayseri, Turkey.; 2 Medical Faculty, Department of Sports Medicine, Erciyes University, Kayseri, Turkey.

**Keywords:** power, lactate concentration, training, combat sports, Wingate test

## Abstract

This study examined the effects of different inspiratory muscle training (IMT) on lactate concentration and utilization during high-intensity exercises. Participants were divided into the following three groups: a chronic inspiratory training group (CRG), an acute inspiratory training group (ARG), and a control group (CG). Participants in the CRG accomplished IMT at an exercise intensity of 60–90% of the maximum inspiratory pressure (MIP) with 30 breaths twice a day for 8 weeks, and those in the ARG performed IMT at an intensity of 40–50% of the MIP for approximately 20 min in the pre-training warm-up phase three times a week. Body composition tests, maximum oxygen uptake, anaerobic power tests, and lactate concentrations of participants were evaluated before and after the intervention. Three consecutive anaerobic power tests (Wingate) were performed to observe changes in lactate concentration and utilization during high-intensity exercises. Blood lactate concentrations were measured immediately at the end of each anaerobic power test, after a 75-s rest, and during passive rest at the 2^nd^, 3^rd^, 4^th^, 5^th^, 10^th^, and 15^th^ min of recovery. The results of the analysis of variance (ANOVA) revealed a significant decrease in blood lactate concentration only in the CRG immediately after the first Wingate test, whereas significant differences in the ARG and the CG were observed after the third Wingate test. After the last Wingate test, a significant decrease was observed after 5 min of recovery in the CRG and after 10 and 15 min of passive rest in both training groups. Herein, we conclude that IMT decreases blood lactate concentration after intense exercise and accelerates lactate utilization during recovery.

## Introduction

Prolonging the duration of high-intensity activities increases the workload on the muscles and induces fatigue in the respiratory muscles, leading to vasoconstriction of peripheral muscle blood vessels and a subsequent decrease in respiratory performance (Lorca-Santiago et al., 2020; Romer and Polkey, 2008; Wells and Norris, 2009). This decrease in respiratory performance reduces exercise tolerance and overall performance. Consequently, the anaerobic glycolytic pathway is activated, leading to lactic acid production from the muscles to meet the elevated workload. The subsequent production of lactic acid contributes to an increase in the blood lactate concentration to 2–4 mmol/L. Continuing exercise at the same intensity results in an inability to effectively neutralise lactic acid, thus reaching the lactate threshold. Reaching the lactate accumulation threshold induces early fatigue by limiting glycolysis and fatty acid oxidation (Çeçen Aksu et al., 2008). Various studies have been conducted to delay fatigue and lactate accumulation thresholds through inspiratory muscle training (IMT) (Rożek-Piechura et al., 2020; Segizbaeva et al., 2015). IMT affects respiratory mechanics by reducing respiratory frequency and increasing exercise tolerance (Archiza et al., 2018; Bailey et al., 2010). Moreover, high-intensity exercises increase blood circulation in extremity muscles, thereby increasing the oxygen supply (Archiza et al., 2018). Furthermore, in addition to athletes' training, IMT reduces lactate concentration and increases athletes' endurance by mitigating early fatigue (Archiza et al., 2018; Guy et al., 2014; Mickleborough et al., 2010; Romer and Polkey, 2008). Inspiratory muscle strengthening reduces lactate production by these muscles and decreases blood lactate concentration (Brown et al., 2008). Fatigue in the inspiratory muscles during exercise limits their optimal functioning and leads to insufficient oxygen supply to the working muscles (De Sousa et al., 2020).

In combat sport matches, athletes should use their energy effectively for the last period of the combat, which generally includes decisive attacks and cardiovascular workload reaches high levels (Slimani et al., 2018). Taekwondo is a traditional martial art included in the Olympics that requires complex motor skills, tactical excellence, and high-level fitness. Competitions are held in three rounds, each lasting 2 min, with a one-minute rest period between the rounds (Ouergui et al., 2021). A single athlete can participate in six or seven bouts of combat on the same day at national or international competitions. During Taekwondo competitions, athletes' heart rates can reach their maximum levels. Depending on the athlete's sex, age, and training level, blood lactate concentrations can reach 11.4 mmol/L or higher (Markovic et al., 2008). Additionally, as in other martial sports, the athletes' minute heart rate and blood lactate concentrations increase with each round of the bouts (Bürger-Mendonça et al., 2015; Folhes et al., 2023). Therefore, reducing acidosis and increasing lactate utilization during rest intervals is essential. Studies on IMT in athletes have been conducted in sports based on aerobic and anaerobic energy systems, such as basketball and football, in which active rest periods are present (Archiza et al., 2018; Goosey-Tolfrey et al., 2010). However, several martial sports, including taekwondo, boxing, kickboxing, wus-hu, and muay-thai, have predetermined round duration, rest periods between rounds, and total competition duration. Most martial sports require high-intensity activities throughout the competition and the dominance of the anaerobic energy system. Athletes in these sports participate in multiple bouts of combat during the day, and generally, the intensity increases with an increasing number of subsequent bouts. The time between bouts played on the same day decreases, especially as the final bouts approach, not allowing athletes to rest and recover.

Existing studies on the impact of IMT on athletes have been conducted in two ways. The first was through chronic IMT performed at a specific intensity using a threshold device, with two sessions per day (morning and evening) and 30 breaths per session. The second way consisted of acute IMT performed before training to warm up the inspiratory muscles which play a smaller role during exercise (Cirino et al., 2023; Diego Fernández-Lázaro et al., 2021; Lorca-Santiago et al., 2020). In our study, IMT during the specific warm-up phase of training to empower athletes to adapt to variations in inspiratory frequency and depth during the warm-up stage of their training was employed, what distinguishes this study from the existing research. We aimed to investigate the effects of different IMT methods (acute and chronic) on lactate concentration and utilization resulting from high-intensity exercises.

## Methods

### Participants

A two-way mixed analysis of variance (ANOVA) necessitated a priori sample size calculation using G*Power (version 3.1). The following parameters were used: statistical test = ANOVA: Repeated measures, within-between interaction; effect size (f) = 0.25; level of significance (α err prob) = 0.05; statistical power (1 − β err prob) = 0.80; number of groups = 3; number of measures = 2; and correlation among repeated measures = 0.50. Based on these parameters, the required sample size was determined to be 42 participants. Men with a minimum of three years of experience in taekwondo, aged between 18 and 30 years, with no history of cigarettes, alcohol, or drug addiction, and who had participated in at least one national competition, were included in this study. Participants were randomly assigned to one of the three groups (a chronic inspiratory training group [CRG], an acute inspiratory training group [ARG], and a control group [CG]) to ensure no significant difference in pre-test performance existed between the groups (Table 1).

**Table 1:** Physical characteristics of participants.

Group		Age (year)	Height (cm)	Body Weight (kg)	BMI	Fat (%)	FFM (kg)
CRG (n = 14)	Pre-Test	19.07 ± 1.97	174.85 ± 7.45	68.80 ± 7.96	21.95 ± 2.36	10.44 ± 5.69	61.45 ± 6.59
	Post Test			69.66 ± 8.95	21.95 ± 2.16	10.44 ± 5.12	62.20 ± 6.56
ARG (n = 14)	Pre-Test	19.50 ± 2.13	173.42 ± 8.95	67.07 ± 7.61	21.82 ± 1.62	9.90 ± 4.09	59.50 ± 8.78
	Post Test			67.17 ± 7.82	21.82 ± 1.70	9.90 ± 3.84	56.66 ± 6.80
CG (n = 14)	Pre-Test	18.71 ± 0.99	175.35 ± 7.30	67.56 ± 10.14	22.10 ± 3.51	11.13 ± 5.15	59.77 ± 7.04
	Post Test			67.22 ± 9.77	22.10 ± 3.29	10.48 ± 4.11	59.58 ± 7.14

BMI: Body mass index, FFM: Fat Free Mass

There were no statistically significant differences found within groups and between groups in terms of age, height, body mass index, body fat percentage, and fat free mass in the pre-tests and post-tests of the participants.

**Figure 1:**
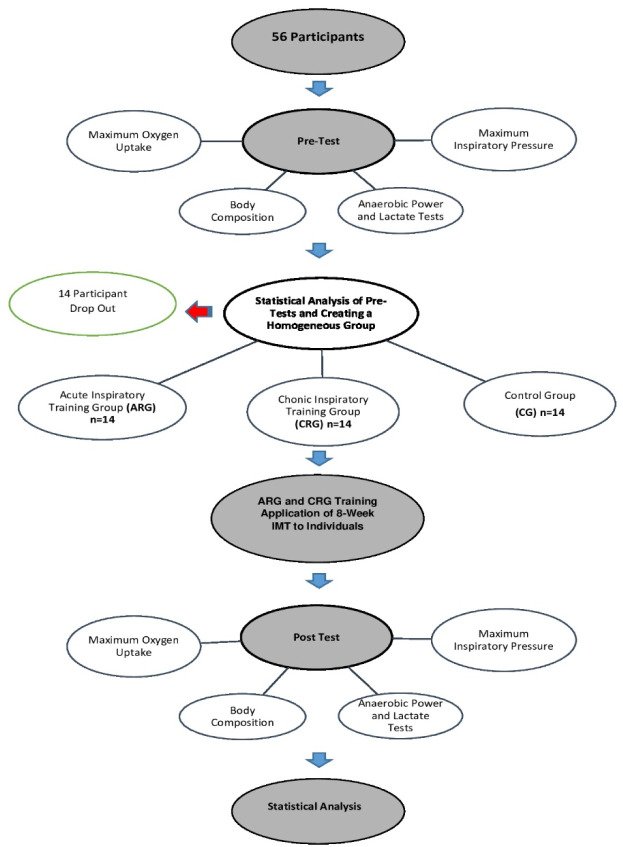
Study design.

All tests included in this study were administered under the supervision of a physician at the Department of Sports Medicine. The daily calorie intake of athletes was monitored for 48 h before testing using the BeBiS 7.2 software (Nutrition Information System, Istanbul, Turkey) to ensure that their performance was not affected by possible variations in nutritional status. Participants were instructed to refrain from exercising for 24 h before each trial and abstain from food intake for 3 h before the test sessions. All measurements were recorded twice: pre-test and post-test. The overall study design is illustrated in Figure 1.

All athletes provided written informed consent for participation in this study. The study protocol was approved by the ethics committee of the Erciyes University Clinical Research (approval number: 2021/33 dated 06 January 2021). The study was conducted in accordance with the tenets of the Declaration of Helsinki.

### Design and Procedures

Each athlete in the experimental group was given the POWERbreathe Medic Plus Heavy inspiratory training device (POWERBreathe International, Ltd., Southam, Warwickshire, England, UK) designed specifically for athletes and intended to be used exclusively by athletes for IMT. Athletes used this device to breathe, with their noses closed using a clamp. This device has a pressure threshold that can be manually adjusted to allow inspiration when a specific pressure level is reached. The POWERbreathe Medic Plus Heavy Resistance training device does not have any specific pressure level during expiration. The intensity level used in this device was predetermined for each individual based on their S-index measured using the POWERbreathe K5 device. The S-index was used to assess inspiratory muscle strength using the POWERbreathe K5 inspiratory muscle trainer. This index estimates an individual's maximum inspiratory pressure (MIP) expressed in cmH_2_O (Minahan et al., 2015; Silapabanleng et al., 2021). The graduated pressure level of the POWERbreathe Medic Plus Heavy Resistance training device was adjusted to the same intensity level as the determined MIP of the athlete.

**Figure 2:**
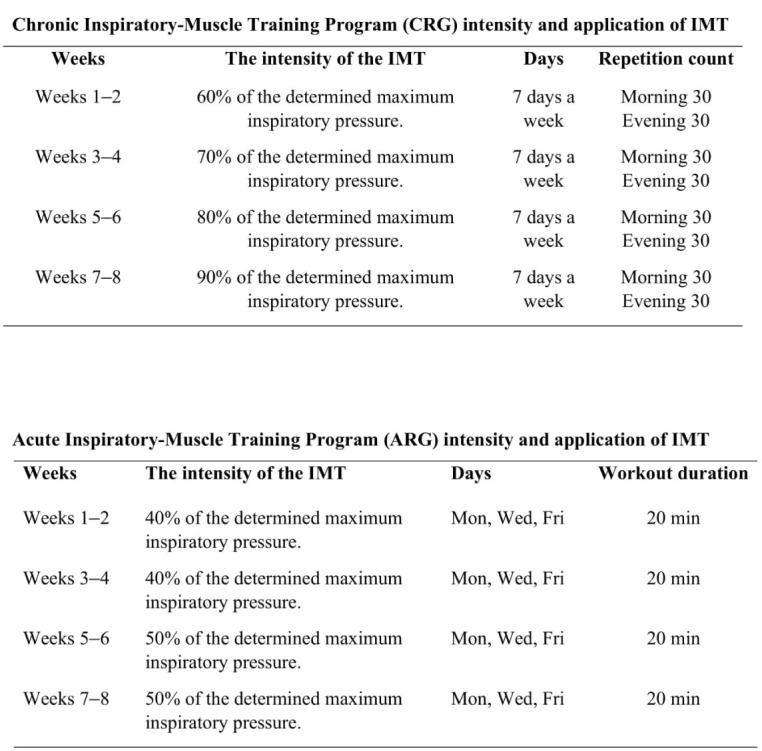
Acute inspiratory-muscle training program (ARG) and chronic inspiratory-muscle training program (CRG) intensity and application of IMT.

Figure 2 illustrates the IMT module for the CRG and the ARG. Participants in the CRG were subjected to IMT twice daily for 30 breaths for 8 weeks (Hartz et al., 2018), whereas IMT was performed three times a week for 8 weeks in the ARG, during the routine warm-up phase before training sessions, for approximately 20 min each time. Although the intensity of this method was low, the loading intensity during the warm-up phase occasionally increased. As the loading intensity increased, the depth and frequency of respiration increased. Despite the low pressure, due to this increase, IMT led to greater effectiveness of training. The conditions of athletes were measured during periods of increased loading using the Borg Scale, which is an indicator of the perceived level of difficulty (Wilson and Jones, 1991). According to the Borg's scale, the intensity of the loads has continued to be difficult and exceedingly difficult (Yaslı et al., 2020).

### Measures

#### Body Composition and the Heart Rate

Body composition was determined using Tanita BC418 (Tanita BC418-Japan). Tanita BC 418 is a professional body composition analyser that uses bioelectrical impedance analysis (BIA) at 50 kHz, equipped with an 8-electrode segmental BIA system, 0.1 kg precision, and the capability for organ fat level analysis with a fat measurement range of 1–75%. The heart rate was measured using a Polar H10 device (Polar H10, Finland). The Polar H10 is designed for high-precision heart rate monitoring and features wireless communication with various compatible devices via Bluetooth and ANT+. This also provides a reliable electrode design for accurate measurements. Using this device, the heart rate was recorded at rest and during exercise (Schaffarczyk et al., 2019).

#### Maximum Oxygen Uptake (VO2max Test)

VO_2max_ values were determined using a cardiopulmonary exercise test battery (Cosmed Quark PFT-Ergo, Rome, Italy) that measured breath-by-breath respiratory gas changes during exercise on a treadmill (h/p/Cosmos Quasar-med, Nussdorf-Traunstein, Germany). Before each test, the flow sensor and gas analyzer components were calibrated according to the manufacturer's recommendations. Athletes performed a warm-up for 10 min on a treadmill, followed by stretching exercises. During testing, the treadmill speed and inclination were increased every 3 min. Athletes experiencing chest pain, dizziness or nausea and unable to continue the test were allowed to stop. The face was covered with an appropriate mask to block the entry of outside air. A wireless receiver was placed on the chest to monitor the heart rate (Polar, Finland). A belt was fastened around the waist to ensure safety. The test was initiated on the monitor after athletes stepped on the treadmill. According to the Bruce protocol, the inclination and speed were increased every 3 min. During this time, the air inhaled and exhaled by the athlete was automatically monitored and displayed on the screen. Achieving the maximal heart rate during testing was determined based on the following criteria: the ratio of expired carbon dioxide (VCO_2_) to oxygen uptake (VO_2_) exceeding > 1.10, expressed as the instantaneous respiratory exchange ratio (RER), and maintaining a plateau in oxygen uptake despite an escalation in exercise intensity. Meeting a minimum of two of these criteria is indicative of attaining VO_2max_. The highest oxygen uptake value, at which at least two of these criteria occurred simultaneously, was considered VO_2max_. Computed VO_2max_ was obtained as the output after the test (Kotte et al., 2015).

#### Maximum Inspiratory Pressure

The S-index of athletes was determined using the POWERbreathe K5 device, which provides a more specific evaluation of inspiratory muscle strength and yields reliable results (Areias et al., 2020). Because this is a pressure-threshold device that requires continuous application of inspiratory pressure, the inspiration adjustment valve was kept open (Volianitis et al., 2001).

During the measurements, athletes were seated comfortably on a chair, and the inspiration device was inserted into the mouth, ensuring no space around the lips. The nose was then closed with a clip. After eight breaths by the participant using the device, the results were automatically collected from the POWERbreathe K5 device, which was linked to a computer (Silva et al., 2018).

#### Anaerobic Power Test (Wingate Test)

A calibrated bicycle ergometer connected to a computer with compatible software was used for anaerobic power testing (Monark 894 E, Sweden). Before the test, the weight to be applied as resistance was automatically calculated based on the athlete's body weight (0.075 kg/kg) using software and placed on the scale. After measuring the clearance of the scale, the athlete was instructed to pedal at maximum speed. The weight on the scale was automatically loaded when the pedal speed reached 60 rpm. Athletes were then asked to pedal at maximum speed for 30 s, and the test was terminated after 30 s. Peak power was automatically calculated by the computer software (Boraczyński and Urniaż, 2008). The fatigue index was calculated using the formula (peak power – minimal power) × 100/peak power (Dotan, 2006). This test was performed three times, with 30 s of exercise and a 75-s rest interval between subsequent bouts.

#### Lactate Evaluation

The lactate threshold values of athletes were determined using a Lactate SCOUT 4 device. Lactate SCOUT 4 is one of the most advanced handheld lactate analyzers, requiring only 0.2 µL of capillary blood and yielding results within 10 s. The device features an integrated step-test function and Bluetooth® connectivity (Tanner et al., 2010). The initial lactate concentration was measured immediately after the 30-s bicycle ergometer test within the first 10 s of completing the test. The second measurement was taken immediately after a 75-s rest interval to determine lactate utilization. This measurement cycle was repeated three times, with the 30-s bicycle ergometer test and a 75-s rest interval between trials. In addition, blood lactate concentration was evaluated at the 2^nd^, the 3^rd^, the 4^th^, the 5^th^, the 10^th^, and the 15^th^ min of recovery.

### Statistical Analysis

All statistical analyses were performed using the IBM SPSS Statistics software version 25.0 for Windows (IBM Corp., Chicago, IL, US). The normality of the distribution was assessed using the Shapiro-Wilk test. Data are presented as mean ± standard deviation. As a statistical comparison test, a two-way mixed analysis of variance (ANOVA) was performed to repeated measurements to determine the main effects and changes in the data with significant interactions over time. To reveal the changes in the variables of which group and time interactions were significant over time, the Bonferroni test was applied, and the group and time changes were compared. A one-way ANOVA was used to reveal statistically significant differences between the groups in the variables of whch group × time interaction was not significant and group differences were significant. The inter-group differences from the results that were significant in one-way ANOVA were determined using the Tukey's post-hoc test (owing to the comparable group sizes and homogeneity of variances). A paired simple test was used to compare the pre- and post-tests within the group. By calculating Cohen’s *d*, effect sizes (ESs) were determined for the changes over time for each variable, which were interpreted as 0 ≤ 0.2 trivial, 0.2 ≤ small < 0.5, 0.5 ≤ moderate < 0.8, 0.8 ≤ large ≤ 1.3, and very large ≥ 1.3 (Cohen, 2013). In addition, the ESs of the time, group, and group × time effects were calculated using partial η2. ES obtained from η2 was considered large if ≥ 0.14, moderate if ≥ 0.06, and small if < 0.06. A *p*-value < 0.05 indicated statistical significance.

## Results

Table 2 summarises the variables measured in MIP. A significant difference was observed in the eight-week IMT time factor and interaction effect (group × time). After eight weeks of IMT, the MIP values increased by 11% and 16% in the CRG and the ARG, respectively.

**Table 2:** Maximum inspiratory pressure results of particular groups.

	CRG (n = 14)			ARG (n = 14)			CG (n = 14)		ANOVA *p*										
	Pre	Post	Pre		Post	Pre		Post	Time			Group				Time*Group			
									F	*p*	η2	F	*p*	η2	F		*p*	η2
MIP cmH_2_O	137.16± 15.25	152.25± 14.65	141.83± 23.06		164.26±23.30	137.30± 21.60		142.52± 24.87	75.84	**<0.001**	0.66	1.52	0.23	0.07	9.29		**0.001**	0.32
	***p* < 0.001**			***p* < 0.001**			*p* = 0.07											

MIP (H_2_O): maximum inspiratory pressure. Significant differences are indicated in bold.

VO_2max_ results indicate a significant difference in the time factor and interaction effect (group × time) (Table 3). After eight weeks of IMT, VO_2max_ values increased by 6.74% and 8.47% in the CRG and the ARG, respectively.

**Table 3:** Comparison of VO_2max_ values of the groups.

	CRG (n = 14)		ARG (n = 14)		CG (n = 14)		ANOVA *p*								
	Pre	Post	Pre	Post	Pre	Post	Time			Group			Time*Group		
							F	*p*	η2	F	*p*	η2	F	*p*	η2
VO_2max_	47.38± 3.18	50.57± 3.66	47.11± 3.00	51.1± 3.17	47.61± 2.92	47.73± 2.74	38.05	**<0.001**	0.49	1.34	0.27	0.06	9.32	**<0.001**	0.32
	***p* < 0.001**		***p* < 0.001**		*p* = 0.87										

VO_2max_: maximum oxygen uptake (ml/kg/min). Significant differences are indicated in bold.

Table 4 summarises the anaerobic power test results of three different Wingate tests. A significant difference was observed only in the time factor of the anaerobic peak power values obtained in the first and second Wingate tests. However, both the time and group factors were statistically significant in the third Wingate test. Inter-group comparisons revealed a difference between the post-test values in the ARG and the CG.

**Table 4:** Comparison of groups’ anaerobic peak power values.

	CRG (n = 14)		ARG (n = 14)		CG (n = 14)		ANOVA p									
	Pre	Post	Pre	Post	Pre	Post	Time				Group			Time*Group		
							F		*p*	η2	F	*p*	η2	F	*p*	η2
1.WPP	757.27± 80.43	820.74± 94.09	748.62± 123.08	819.21± 110.70	745.53± 131.72	764.96± 132.74		23.65	**<0.001**	0.38	0.39	0.68	0.02	2.31	0.11	0.11
	***p* = 0.003**		***p* = 0.015**		***p* = 0.018**											
2.WPP	653.56± 94.43	682.23± 111.44	676.51± 107.13	710.32± 93.50	633.78± 141.41	652.81± 165.20		5.83	**0.021**	0.13	0.65	0.53	0.03	0.15	0.86	0.01
	*p* = 0.17		*p* = 0.18		*p* = 0.18											
3.WPP	532.72± 136.95	565.85± 79.92**^a,b^**	573.92± 101.75	630.84± 78.60**^a^**	486.34± 90.99	510.17± 102.12**^b^**		9.62	**0.004**	0.20	4.48	**0.018**	0.19	0.66	0.53	0.03
	*p* = 0.14		***p* = 0.04**		*p* = 0.17											

WPP: Wingate test anaerobic peak power (watt). Significant differences are indicated in bold.

a, b: there is no difference between groups carrying the same symbol in the same row.

Blood lactate concentrations obtained at different time points are shown in Table 5. Significant statistical differences were found in blood lactate concentrations for the following time factors: resting blood lactate levels, immediately after the first Wingate test, and the second minute after completing the Wingate tests. A statistically significant group × time difference was observed in blood lactate values obtained 5 min after the completion of Wingate tests. Moreover, a statistically significant difference in the time factor and the group-time interaction was observed in the 10^th^ and the 15^th^ min of recovery.

**Table 5:** Comparison of groups' blood lactate concentrations (mmol/l).

	CRG (n = 14)		ARG (n = 14)		CG (n = 14)		ANOVA *p*								
	Pre	Post	Pre	Post	Pre	Post	Time			Group			Time*Group		
							F	*p*	η2	F	*p*	η2	F	*p*	η2
Rest	1.35± 0.30	1.10± 0.41	1.21± 0.44	1.15± 0.34	1.40± 0.33	1.20± 0.25	10.52	**0.002**	0.21	0.56	0.58	0.03	1.13	0.33	0.06
	***p* = 0.020**		*p* = 0.59		***p* = 0.006**										
1.Win. afterwards	8.64± 5.28	5.47± 3.45	7.17± 4.78	5.96± 2.79	7.76± 1.86	7.37± 2.36	8.29	**0.006**	0.18	0.35	0.71	0.02	2.22	0.12	0.10
	***p* = 0.011**		*p* = 0.30		*p* = 0.53										
75-s rest	12.57± 3.66	12.05± 3.80	12.37± 4.87	11.65± 4.97	12.82± 4.19	13.26± 3.31	0.17	0.68	0.00	0.30	0.75	0.02	0.31	0.73	0.02
	*p* = 0.69		*p* = 0.61		*p* = 0.27										
2.Win. afterwards	14.62± 4.99	13.82± 3.45	13.37± 5.51	14.38± 3.33	15.29± 4.16	16.65± 4.12	0.49	0.49	0.01	1.36	0.27	0.07	0.79	0.46	0.04
	*p* = 0.54		*p* = 0.54		*p* = 0.18										
75-s rest	16.38± 3.18	15.3 ± 2.66	15.18± 3.78	15.47± 3.56	17.35± 2.97	17.02± 1.77	0.50	0.48	0.01	1.85	0.17	0.09	0.58	0.56	0.03
	*p* = 0.31		*p* = 0.78		*p* = 0.48										
3.Win. afterwards	17.17± 5.05	15.09± 2.95^a,b^	15.50± 3.79	14.32± 3.22^a^	18.00± 2.42	17.61± 2.92^b^	3.77	0.06	0.09	3.65	**0.035**	0.16	0.61	0.55	0.03
	*p* = 0.16		*p* = 0.35		*p* = 0.37										
2. min	19.36± 3.75	16.69± 2.42	17.37± 2.76	15.78± 2.26	18.22± 3.42	17.15± 3.15	10.18	**0.003**	0.21	1.38	0.27	0.07	0.72	0.49	0.04
	*p* = 0.06		*p* = 0.09		*p* = 0.11										
3. min	17.53± 3.09	16.55± 1.14	16.22± 1.98	16.32± 2.37	18.24± 2.49	16.97± 3.09	2.98	0.09	0.07	1.47	0.24	0.07	1.03	0.37	0.05
	*p* = 0.19		*p* = 0.90		***p* = 0.042**										
4. min	17.58± 3.37	16.25± 2.07	16.66± 4.37	16.60± 2.41	17.37± 2.88	15.98± 2.13	3.06	0.09	0.07	0.06	0.95	0.00	0.68	0.52	0.03
	*p* = 0.09		*p* = 0.96		***p* = 0.048**										
5. min	17.89± 3.41	15.62± 2.79	15.90± 2.26	14.94± 2.10	16.71± 3.03	17.66± 3.71	3.08	0.09	0.07	1.76	0.19	0.08	4.70	**0.015**	0.19
	***p* = 0.004**		*p* = 0.21		*p* = 0.21										
10. min	18.05± 3.36	15.68± 2.84	16.66± 2.14	15.06± 2.29	17.49± 2.38	17.29± 2.59	28.70	**<0.001**	0.42	1.35	0.27	0.07	5.98	**0.005**	0.24
	***p* < 0.001**		***p* = 0.001**		*p* = 0.66										
15. min	17.10± 3.27	13.7± 2.46	15.71± 2.10	12.42± 1.35	16.29± 2.79	15.70± 3.05	35.45	**<0.001**	0.48	2.76	0.08	0.12	5.04	**0.011**	0.21
	***p* < 0.001**		***p* < 0.001**		*p* = 0.40										

Win.: Wingate anaerobic test. Significant differences are indicated in bold.

a.b: There is no difference between groups carrying the same symbol in the same row.

## Discussion

This study examined the effects of two IMT methods on lactate concentration and utilization time during high-intensity exercises. MIP and VO_2max_ values showed significant improvement in both training groups after the 8-week training intervention. A significant difference in the blood lactate concentrations measured at different time points was observed between the two training methods. Lactate levels showed a significant decrease in both training groups at the 10^th^ and the 15^th^ min following the three Wingate tests. However, only the CRG demonstrated a significant decrease in measurements taken immediately after the first 30-s Wingate test. A significant difference was observed only in the ARG after the third Wingate test, and a significant decrease was observed at 5^th^ min of recovery only in the CRG. The addition of IMT to taekwondo athletes' routines accelerated lactate utilization during passive recovery at the 5^th^, the 10^th^, and the 15^th^ min after repeated intense exercises.

Studies analysing the effects of IMT exercises on blood lactate concentration and utilization are limited (Fernández-Lázaro et al., 2021). These studies report that IMT reduces lactate concentration and accelerates lactate utilization, depending on the intensity of the exercise. In contrast, few studies have reported that IMT has no significant impact on blood lactate production or utilization. The results vary depending on the intensity, the type, and the method of IMT (Brown et al., 2012; Chiappa et al., 2008, 2009; Johnson et al., 2012; McConnell and Sharpe, 2005; Najafi et al., 2019). This study aimed to investigate the effects of IMT using different methods during repeated high-intensity activities, which differs from previous studies.

Studies on the effects of IMT in martial arts are relatively scarce. In judo, where inspiratory muscle pre-activation practices are performed, the results of the Special Judo Conditioning Test clarify that it has an impact on technical tactics (obtained through notation analysis of video recordings, such as the number of attacks, bout quality, and average bout duration) (Cirino et al., 2021). No significant effects were observed in the respiratory variables among mixed martial arts and kickboxing athletes following IMT (Alnuman and Alshamasneh, 2022). The 8-week IMT conducted on middle-aged taekwondo athletes classified as smokers showed significant changes in body composition, muscle strength, muscle endurance, flexibility, cardiopulmonary function, respiratory function and isokinetic muscle function (An et al., 2017). However, there is no evidence in those studies regarding physiological variables such as resting blood lactate and post-exercise blood lactate concentrations.

In this study, a significant decrease in lactate concentration was observed only in the CRG immediately after the first Wingate test, which demonstrated that the high-intensity exercise applied in the CRG delayed lactate accumulation that occurs during high-intensity exercise. This delay indicated an improvement in the anaerobic capacity of the skeletal muscles. The potential ergogenic effects of IMT, including the improvement in athletic performance by reducing or delaying respiratory fatigue, metabolic reflex mechanism of respiratory muscles, and blood lactate concentration observed in our study were consistent with those of a previous study (Fernández-Lázaro et al., 2021). The significant difference found only between the ARG and the CG immediately after the third Wingate test suggests that the anticipated fatigue in response to consecutive high-intensity exercise was delayed, which indicates the sustainability of the displayed anaerobic performance. This could be attributed to lactate utilization. Conversely, CRG training delayed the onset of lactate accumulation, while ARG training improved lactate utilization and ensured the sustainability of exercise despite its high intensity. The mechanism by which inspiratory muscle endurance training extends the duration of steady-state exercise intensity remains unclear. However, a decrease in blood lactate accumulation during exercise after inspiratory training could be a probable cause. The possible mechanisms by which IMT decreases blood lactate concentration are as follows: (1) working muscles produce less lactate due to reduced respiratory activity and therefore have a lower overall energy demand; and (2) trained respiratory muscles effectively use additional lactate as an energy substrate for their activities (Spengler et al., 1999). Since the heart rate was not decreased during exercise in our study and the decrease in oxygen saturation immediately after the Wingate test indicated a decrease in inspiratory activity (*p* < 0.05), we believe that the second mechanism validates our findings. Spengler et al. (1999) analysed the effects of respiratory endurance training on oxygen uptake and blood lactate concentration during exercise and reported that respiratory endurance training prolonged the duration of steady-state exercise and reduced the blood lactate concentration during exercise. The decrease in lactate concentration was likely due to improved lactate uptake by the trained respiratory muscles.

Another variable that supported our findings was the peak power value obtained from the Wingate tests. Compared to the CG, the ARG showed a significant increase in the peak power value obtained in the third Wingate test. Anaerobic energy production was sustained in the ARG and increased during consecutive executions. Furthermore, IMT did not affect the results of the Wingate anaerobic power test (Johnson et al., 2007). However, the mechanisms underlying the increase in IMT-induced anaerobic energy remain unknown. The difference between the ARG and the CG could be explained by the fact that a delay in the accumulation of metabolites partially delayed fatigue caused by acute IMT, when compared to chronic IMT (Johnson et al., 2007).

A significant decrease in lactate concentration in the 5^th^ min after the completion of the three Wingate tests was observed only in the CRG, indicating that CRG training accelerated recovery after exercise. Both training groups showed a significant decrease in the 10^th^ and the 15^th^ min of recovery, highlighting the effectiveness of our training methods during recovery. Another variable reflecting the impact of our training on recovery was the heart rate measured after the Wingate test. After the Wingate tests, all three groups showed a significant decrease in the 2^nd^ min, representing the effect of specific training on branch exercises. However, only our intervention groups showed a significant decrease in the 3^rd^, the 10^th^, and the 15^th^ min of recovery, supporting the impact of our IMT on recovery. Although the CRG recovered more quickly in terms of lactate concentration after the three Wingate tests, a significant reduction in the heart rate in the 4^th^ and the 5^th^ min was observed only in the ARG. Therefore, IMT improves respiratory functions; the inverse correlation between blood flow to the muscles triggers respiratory function during exercise to increase the perfusion of skeletal muscles and VO_2max_ (Harms et al., 1997).

The rate of muscle glycogen use is high during high-intensity physical activity, which rapidly increases lactic acid production because oxidative processes in the Krebs cycle exceed mitochondrial capacity. However, with the improvement in aerobic capacity gained after IMT, the amount of oxygen delivered to the muscles increases, thereby delaying the lactate threshold. Therefore, a significant reduction in lactate levels is associated with improved VO_2max_ (Fernández-Lázaro et al., 2021; McConnell and Sharpe, 2005).

During intense exercise, respiratory muscles can utilise 16% of the cardiac output and decrease oxygen availability for the skeletal muscles responsible for movement. This indicates that the respiratory system is a limiting factor for physical activity performance and VO_2max_. However, adequate IMT can improve the fatigue tolerance of the respiratory muscles, enhance respiratory efficiency, and delay the metabolic reflex mechanism of the respiratory muscles. Conversely, IMT enhances lactate reduction, VO_2max_, and aerobic metabolism (Fernández-Lázaro et al., 2021). In our study, VO_2max_ improved significantly in both inspiratory training groups. The increase in VO_2max_ was significantly greater in the ARG than in the CG.

IMT performed with the POWERbreathe device results in significant improvements in the oxidative capacity of the diaphragm and MIP, which improves strength and enhances resistance to fatigue. A change in MIP of >25% after IMT suggests an improvement in endurance. Additionally, improvements ranging from 6.8% to 21.5% contribute to athletic endurance (Fernández-Lázaro et al., 2021). In our study, significant improvements in MIP were observed only in the experimental groups (15.8% and 11% in the ARG and the CRG, respectively). Considerable improvements in MIP are observed in training programmes of longer duration, such as of 12 weeks (Fernández-Lázaro et al., 2021). However, in our study, where both training groups implemented an 8-week training programme, the percentage of improvement in MIP was higher in the acute training method (Archiza et al., 2018; Edwards et al., 2008; Griffiths and McConnell, 2007; Hartz et al., 2018; Koç and Saritas, 2019).

Studies investigating the effects of IMT using POWERBreathe on VO_2max_ and MIP (Archiza et al., 2018; Edwards et al., 2008; Griffiths and McConnell, 2007; Hartz et al., 2018; Koç and Saritas, 2019) have documented that IMT exercises increase VO_2max_ and MIP values.

In contrast, few studies have reported that IMT does not affect VO_2max_ (Amonette and Dupler, 2002; Riganas et al., 2008), which may be due to the variations in IMT devices or IMT methods applied.

## Conclusions

In this study, we investigated the effects of two IMT interventions on lactate concentration and utilization during high-intensity exercise in taekwondo athletes. The addition of IMT to athletes' usual training increased inspiratory muscle strength and maximal oxygen uptake. Contrary to many previous studies that demonstrated no effects of IMT on the Wingate anaerobic power test outcomes, the two interventions in this study influenced the peak power values obtained from the Wingate test. Furthermore, IMT reduced the blood lactate concentration during intense exercise and accelerated lactate utilization during recovery. The chronic intervention delayed the onset of lactate accumulation, whereas the acute intervention improved lactate utilization and therefore enhanced exercise endurance during high-intensity exercise. The use of IMT devices in routine training, such as the POWERbreathe, which are portable, easy to use, and relatively inexpensive compared to laboratory equipment, considerably impacts performance of martial arts athletes. IMT is recommended for Taekwondo athletes to improve both their aerobic and anaerobic power and capacity. Furthermore, our interventions had a significant effect on lactate levels, which are crucial for improving performance in the taekwondo-kyorugi competitions. The chronic inspiratory training method can be recommended for athletes prone to early fatigue, while the acute inspiratory training method can be recommended for those who intend to prolong their resistance to fatigue.t
